# Sialyl-Tn expression correlates with reduced c-Myc and immune modulation in triple negative breast cancer

**DOI:** 10.1038/s41598-025-21496-3

**Published:** 2025-10-28

**Authors:** Rita Adubeiro Lourenço, Daniela Ferreira Barreira, Carla Lopes, Pedro Granjo, Ana Sofia Rodrigues, Zélia Silva, Manuela Martins, Ana Rita Grosso, Paula A. Videira

**Affiliations:** 1 UCIBIO – Applied Molecular Biosciences Unit, Department of Life Sciences, NOVA School of Science and Technology | FCT NOVA, Universidade NOVA de Lisboa, Caparica, 2829 − 516 Portugal; 2 Associate Laboratory i4HB - Institute for Health and Bioeconomy, NOVA School of Science and Technology | FCT NOVA, Universidade NOVA de Lisboa, Caparica, 2829 − 516 Portugal; 3 CDG & Allies – Professionals and Patient Associations International Network (CDG & Allies – PPAIN), Caparica, 2829 − 516 Portugal; 4 Centro Hospitalar Universitário de Lisboa Central, EPE e Serviço de Anatomia Patológica, Lisboa, 1150 − 199 Portugal

**Keywords:** Triple negative breast cancer, Sialyl-Tn antigen, ST6GalNAcI, ST6GALNAC1 gene, C-Myc, Immune evasion, Targeted therapies, Breast cancer, Cancer microenvironment, Tumour biomarkers, Data acquisition, Databases, Gene ontology, Statistical methods, Immune evasion, Biomarkers, Cancer

## Abstract

Triple negative breast cancer (TNBC) is an aggressive, heterogeneous cancer with lack of targeted therapies. The cancer-associated sialyl-Tn (STn) antigen has a significant role in cancer, yet its involvement in TNBC remains unexplored. This study investigates STn’s role in TNBC, analysing its expression in the primary tumour tissues of 126 TNBC patients alongside other biomarkers and clinical features. STn was detected in 23.8% of cases, exhibiting significantly reduced survival and lower c-Myc expression. Additionally, data from The Cancer Genome Atlas (TCGA) TNBC cohort confirmed this association, showing that high levels of *ST6GALNAC1*, gene encoding the enzyme responsible for the STn synthesis, were inversely correlated with *MYC* expression and positively associated with TGF-β signalling genes and immunosuppressive cell infiltrates, such as macrophages M2 and regulatory T cells. Accordingly, STn-positive TNBC cell lines exhibited increased proliferation and lower c-Myc protein expression, while co-culture with macrophages enhanced M2 polarization. This study discloses, for the first time, a subgroup of TNBC patients expressing the STn antigen, pointing to an immunosuppressive environment that may lead to the observed negative correlation between STn and c-Myc. These results introduce STn as a potential prognostic biomarker and therapeutic target, laying the groundwork for more effective, personalized treatments for TNBC.

**Supplementary Information:**

The online version contains supplementary material available at 10.1038/s41598-025-21496-3.

## Introduction

Triple negative breast cancer (TNBC) is an aggressive subtype, representing 15–20% of all breast cancer cases^1^. Characterised by the absence of hormone receptors and human epidermal growth factor receptor 2 (HER2) expression, TNBC lacks targeted therapies, leaving chemotherapy as the primary treatment^1,2^. Unfortunately, this often leads to poor survival outcomes, underscoring the urgent need to develop targeted treatments for TNBC.

Efforts to improve treatment have focused on categorising TNBC subtypes based on histological and molecular characteristics, using different techniques, including immunohistochemical and gene expression profiling^3,4^. However, a comprehensive understanding of TNBC’s heterogeneity remains elusive, highlighting the critical need for improved stratification. Identifying new biomarkers in TNBC is essential for stratifying patients, predicting therapy responses, and identifying new therapeutic targets.

The tumour microenvironment (TME) is critical in promoting resistance to conventional therapies in TNBC and enhancing malignancy^5^. Immune cells within the TME are crucial in tumour development and control, exhibiting both pro and anti-tumorigenic effects. Particularly important are tumour-associated macrophages (TAMs), commonly found in tumours exhibiting two distinct phenotypes: M1, with pro-inflammatory and anti-tumour responses, and M2, which have immunosuppressive properties that favour tumour growth and malignancy, associated with poorer prognosis^6,7^.

The aberrant sialylation (addition of sialic acid to glycans) of tumour cells has gathered significant interest as a contributor to immune evasion in various cancers^8^. Besides sialyl Lewis X, some sialylated glycans are overexpressed or newly expressed by cancer cells, such as the sialyl-Tn (STn). The STn is a cancer-associated short O-glycan typically absent in normal healthy tissues, which arises from the early sialylation of the Thomsen-nouveau (Tn) antigen due to the overexpression of the enzyme ST6GalNAc-I and/or disruption of the core 1 O-glycan elongation in various cancers, including bladder, colon, ovarian and breast^9–15^. Its expression significantly impacts cell adhesion, migration, invasion, metastasis, and chemotherapy resistance, and promotes immune evasion^16–18^. Furthermore, STn was identified in a subpopulation of TNBC but the clinical features and impact on immune evasion of this subpopulation remained unclear^19^.

This manuscript aims to analyse the biological significance of the STn expression in TNBC and characterize its context and clinical implications. To achieve this goal, we thoroughly analysed a large set of tumour tissue microarrays (TMA) to characterise STn expression in TNBC cancer tissues and establish correlations with clinical features and biomarkers associated with stemness and epithelia-to-mesenchymal transition (EMT). Further, we extended our analysis to a TNBC cohort from The Cancer Genome Atlas (TCGA), investigating links between the *ST6GALNAC1* gene, clinical features, and other gene expression patterns. Furthermore, we investigated the MDA-MB-231 TNBC cell line engineered to express STn. By shedding light on the intricate interplay between STn expression, tumour progression, and immune evasion in TNBC, this study aims to pave the way for targeted therapeutic strategies and personalized treatment approaches tailored to the heterogeneous nature of this aggressive subtype.

## Materials and methods

### Patients and clinical data

Tumour samples were collected from 126 women with histological diagnosis of TNBC at Centro Hospitalar Lisboa Central–Hospital de São José (CHLC-HSJ), Lisbon, Portugal, until 2015 with minimum 5 years follow-up or until death. The study was approved by the CHLC-HSJ Ethics and Investigation Committee (MM/2016) and followed the regulations of Direção Geral de Saúde (Portugal) for ethical research involving human subjects (ref: 09/2013). Per these national regulations, informed consent was not required from patients, as anonymised archival specimens were used. All research was conducted in accordance with relevant guidelines and regulations. Complete information on the HSJ-TNBC cohort is detailed in Supplementary Table S1 and S2.

### TMAs immunohistochemistry

TMAs were prepared from histologically representative areas of each tumour preserved as formalin-fixed paraffin-embedded (FFPE) blocks with a diameter of 3 mm. Optimal antibody dilutions were established according to the College of American Pathologists and Laboratory Quality Centre guidelines^20^, and experimental conditions and control tissues, selected according to the Human Protein Atlas indication, are in Supplementary Table S3 and Fig. S1. Immunohistochemistry of TMA Sect. (4 μm) was done using an automated slide staining system (Ventana Medical Systems, Inc., Oro Valey, AZ, USA). The sections were deparaffinised and submitted to antigen retrieval with customised conditions (Supplementary Table S3). The ultraView Universal DAB Detection Kit (Ventana Medical Systems, Inc.) was used for blocking endogenous peroxidase activity and detection of biomarkers. Counterstain was accomplished with haematoxylin, followed by a bluing reagent (Ventana Medical Systems, Inc.). TMAs were evaluated by two independent observers (CL and MM) and semi-quantitative score evaluation was established by multiplying staining and intensity scores (Table 1). Supplementary Table S1 contains the staining scores of each biomarker analysed.

**Table 1:** Semi-quantitative scoring of the immunohistochemistry evaluation.

Score	Stain of appropriate cells
0	Negative
1	< 25%
2	25–50%
3	51–75%
4	> 75%

Score	Intensity of appropriate cells
0	No stain
1	Weak
2	Intermediate
3	Strong

### Retrieval and selection of data from TCGA and TCIA databases

The breast carcinoma dataset from TCGA (https://cancergenome.nih.gov/)^21,22^ was downloaded from the Genomic Data Commons (GDC) using R v 4.3.2^23^ packages “TCGAbiolinks” v3.19^24,25^ and “SummarizedExperiment” v1.34.0^26^. Data included RNA-Seq as STAR–Counts and clinical data. Using an adaptation of the pipeline previously described^27^, 160 TNBC patients were selected for the TCGA-TNBC cohort. The Cancer Immune Atlas (TCIA) (https://tcia.at/)^28^ was used to predict the cellular composition of immune cell infiltrates of solid tumours in the TCGA-TNBC cohort, characterised using gene set enrichment analysis and RNAseq-based deconvolution.

### Cell culture

The human TNBC cell line MDA-MB-231 wild type (WT) and the stably transfected variant with an ST6GALNAC1 expression vector (STn+) were provided by Professor Philippe Delannoy (University of Lille, France), who produced and characterized them as previously described^29^. Cells were cultured in complete Dulbecco’s modified Eagle medium (cDMEM; Corning, NY, USA), supplemented with 10% (v/v) foetal bovine serum (FBS), 2mM L-glutamine, and 100 g/mL penicillin/streptomycin, all from Gibco/Thermo Fisher Scientific (Waltham, MA, USA), in a humidified incubator at 37 °C with an atmosphere containing 5% CO_2_. Cells were routinely tested for mycoplasma contamination using the MycoAlert™ kit (Lonza, Basel, Switzerland).

### Monocyte isolation and co-culture with MDA-MB-231 WT and STn + cell lines

Peripheral blood mononuclear cells (PBMCs) were isolated from buffy coats obtained from healthy anonymous donors provided by the Instituto Português do Sangue e da Transplantação (IPST), as described previously^30^. The donors had given written and informed consent under the reference IMP.74.52.4, in compliance with Directive 2004/23/EC, which establishes standards for the quality and safety of human tissues and cells (Portuguese Law 22/2007, June 29). This process was carried out with the approval of the ethics committee of IPST 30,072,015. Monocytes were isolated by positive selection using anti-CD14 coated magnetic beads (Miltenyi Biotech, Bergisch Gladbach, Germany) as described^30^ and cultured in complete Roswell Park Memorial Institute (cRPMI)-1640 medium supplemented with 10% (v/v) FBS, 2 mM L-glutamine, 100 g/mL penicillin/streptomycin, 1 mM sodium pyruvate and 1% (v/v) non-essential amino acids, all from Gibco/Thermo Fisher Scientific. The isolated monocytes were co-cultured with the MDA-MB-231 WT and STn + cell lines in a 6-well plate in a 1:2 proportion. A control of monocytes alone was also included. The cells were incubated for 5 days to allow monocyte adherence and differentiation into a macrophage-like phenotype.

### Immunocytochemistry

For immunocytochemistry analysis of STn expression on MDA-MB-231 cell lines, cells were grown to ~ 95% confluency in a monolayer, collected using trypsin + EDTA 1X for the detachment, and fixed in 2% paraformaldehyde (PFA). Cell pellets were washed, embedded in 1% agarose in PBS, and processed for paraffin embedding and sectioning (20 sections, 4 μm) at the Champalimaud Foundation Histopathology facility. The sections were deparaffinised using xylene, hydrated in graded alcohols, and blocked for endogenous peroxidase activity with H_2_O_2_. After antigen retrieval with 0.01 M sodium citrate buffer (pH 6), sections were incubated with Horse Serum (Vector Laboratories, Newar, CA, USA) and incubated overnight with the primary anti-STn antibody (hIgG1 version of L2A5^19^ followed by incubation with biotinylated goat anti-human secondary antibody (Vector Laboratories) for 1 h. Detection was performed by incubation with the ABC reagent (Thermo Fisher Scientific) followed by exposure to a DAB Peroxidase substrate kit (Vector Laboratories). Counterstaining was achieved using hematoxylin. Slides underwent dehydration with decreasing concentrations of alcohol and xylene before mounting.

### Flow cytometry

For flow cytometry analysis of STn expression on MDA-MB-231 cell lines, 1 × 10^5^ cells were incubated with the primary anti-STn antibody (hIgG1 version of L2A5 clone^19^ diluted 1:300 for 30 min, followed by incubation with FITC-conjugated anti-human IgG (Southernbiotech) for 20 min.

For macrophage-like cells differentiation and activation after co-culture analysis, cells were stained with APC-conjugated anti-MHC-II (GRB-a; Immunostep S.L, Salamanca, Spain), PE-conjugated anti-CD163 (RM3/1; BioLegend, San Diego, CA, USA), Alexa Fluor^®^ 488-conjugated anti-CD206 (15 − 2; BioLegend) and PerCP-conjugated anti-CD45 (HI30; BioLegend) for 30 min. Cells were fixed with flow fix 2% paraformaldehyde (Polysciences, Inc., Warrington, PA, USA), and data was acquired in the Attune flow cytometer (Thermo Fisher Scientific, Waltham, MA, USA) and analysed using FlowJo™ v10.8.1 Software (BD Life Sciences, Franklin Lakes, NJ, USA).

### Western blot

Whole-cell lysates were obtained using Pierce™ IP Lysis Buffer (Thermo Fisher Scientific) containing cOmplete™, Mini, EDTA-free Protease Inhibitor Cocktail (Roche, Basel, Switzerland). Protein was quantified with the Pierce™ BCA Protein Assay kit (Thermo Fisher Scientific) following the manufacturer’s instructions. Cell lysates were separated in 10% SDS-PAGE, using 50 µg per lane, and transferred to PVDF membranes (GE Healthcare Life Sciences, Chicago, IL, USA). Blocking was performed with 5% BSA in Tris-buffered saline with 0.05% Tween 20% (TBS-T) for 1 h, followed by incubation overnight with the mouse IgG1 Anti-c-Myc monoclonal antibody (9E10; Santa Cruz Biotechnology, Dallas, TX, USA) diluted 1:1000 and the Anti-α-Tubulin monoclonal antibody (Sigma-Aldrich, St. Louis, MO, USA) diluted 1:2500. For the detection a goat anti-mouse IgG1 horseradish peroxidase-conjugated antibody (Abcam, Cambridge, UK) was diluted 1:2500 for 1 h. The signal was detected using the Lumi-Light Western Blotting Substrate (Roche) and chemiluminescence films (Amersham Hyperfilm™ ECL GE Healthcare Life Sciences). Bands’ signal intensity was quantified using ImageJ software version 1.8.0_172.

### Cell proliferation measurement

To study cell proliferative capacity, cells were labelled with CellTraceTM carboxyfluorescein succinimidyl ester (CFSE) Proliferation Kit (Molecular Probes, Eugene, OR, USA) and resuspended in cDMEM at the final concentration of 1 × 10^6^ cells/mL and incubated with 5 µM CFSE for 48 h following the manufacturer’s instructions. Cell fluorescence was measured by flow cytometry, and the percentage of proliferative and non-proliferative cells was determined as described^31^.

### Statistical analysis

#### Clinical data analysis

The following statistical analysis was performed using R v 4.3.2. The overall survival and progression-free survival rates, studied in the HSJ-TNBC and the TCGA-TNBC cohorts, were established with the Kaplan–Meier method and statistical significance determined by the log-rank test. The R packages “survival” v3.5-8^32^ and the “survminer” v0.4.9^33^ were used. The Fisher exact test was used to compare the frequencies of STn- and STn + groups with the clinicopathological features groups, and the plots generated using the R base packages. Tests were considered statistically significant when *p* < 0.05.

#### Correlation analysis

Normality was tested with the Shapiro–Wilk test and the non-parametric Spearman correlation tests with adjusted p-value by the False Discovery Rate (FDR) method (adj.p-value) was used to identify correlations between: STn and the other biomarkers analysed *in tissue* in the HSJ-TNBC cohort; *ST6GALNAC1* gene and the genes directly responsible for the selected biomarkers; *ST6GALNAC1* gene and the genes of proteins involved in the TGF-β pathway; and between the *ST6GALNAC1* gene and immune infiltrates frequencies in the TCGA-TNBC cohort. Heatmaps were generated using the “corrplot” package v0.92^34^, and correlations were considered statistically significant when adj.*p* < 0.05. The “EnsDb.Hsapiens.v86” R package v2.99.0^35^ was used to obtain the gene names.

#### Comparison between groups

To compare the expression of the biomarkers between STn + and STn- groups, and the gene expression and immune cells infiltrates frequencies between high and low *ST6GALNAC1* gene expression groups (defined by the median expression of the gene), the Wilcox t-test was used, using the R computational language. The p-value was adjusted using the FDR method and considered statistically significant when adj.*p* < 0.05.

Using the GraphPad Prism version 8.0.1 for Windows (GraphPad Software, San Diego, CA, USA, www.graphpad.com), the parametric two-tailed unpaired t-test with Welch’s correction was used to compare the relative protein expression of c-Myc and the non-parametric Mann-Whitney test was used to compare the frequency of proliferative and non-proliferative cells, between the MDA WT and STn + cell lines. The parametric one-way ANOVA and Tukey’s multiple comparisons test were used to compare the mean fluorescence intensity (MFI) and frequency of positive cells. Tests were considered statistically significant when p and adj.*p* < 0.05. All bar plots were generated using GraphPad Prism, and data is presented as mean ± SEM.

#### Gene set enrichment analysis (GSEA)

For the TCGA-TNBC group, we conducted a differential expression analysis comparing high and low *ST6GALNAC1* patients (based on the median expression level of the *ST6GALNAC1* gene), using the “limma” R package v3.56.2^36^. Functional enrichment analysis was performed, utilizing the sign (log2 Fold Change (FC)) - log10(FDR) score for the GSEA carried out through the WEB-based GEne SeT AnaLysis Toolkit (WebGestalt) 2019 (https://2019.webgestalt.org/)^37^, with a q-value of ≤ 0.05 set as the cut-off. We identified non-redundant terms related to Gene Ontology (GO) biological processes, as outlined on their official website. The plot was generated using the “ggplot2” R package v3.4.4^38^.

## Results

### STn expression is associated with poor survival and low Myc levels

To elucidate the implication of STn presence in TNBC, we assessed its expression in a new TNBC cohort enclosing 126 patients from the CHLC-HSJ (complete information detailed in Supplementary Table S1 and S2). The women in the HSJ-TNBC cohort median age at diagnosis was 62 years (Min = 27 years, Max = 90 years), and the median tumour size was 25 mm (Min = 6 mm, Max = 101 mm). The overall survival ranged from 3 months to 22 years and 5 months (Supplementary Fig. S2A), while progression-free survival ranged from 0 to 14 years, with a 50% probability of progression-free survival within the first 8 years post-diagnosis (Supplementary Fig. S1B). Women with age ≥ 62 years, tumours ≥ 25 mm, and metastasis revealed worse progression-free and/or overall survival (Supplementary Fig. S2C-G).

Besides the expression of STn, we also evaluated the expression of the biomarkers CD44, N-Cadherin, β-Catenin, MMP9, Oct4, Sox2, c-Myc, SALL4, and Ki67. Immunohistological analysis revealed heterogeneity in biomarker expression among the TNBC, with STn detected in the membrane and in the cytoplasm of 30 tumours (23.8%) and not in stroma (Supplementary Fig. S2H).

**Fig. 1:**
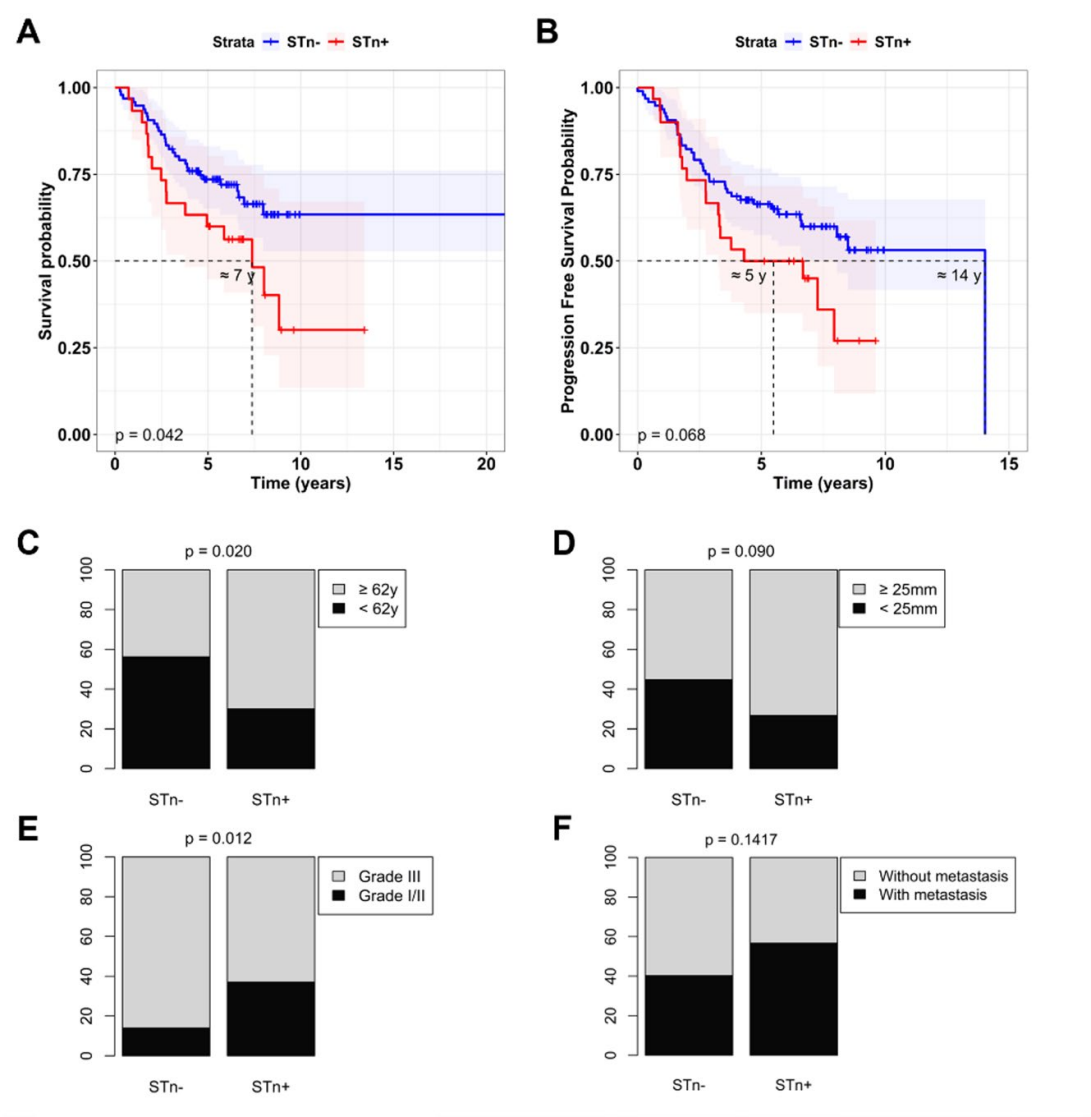
STn expression is associated with clinical features in the TNBC-HSJ Cohort. The Kaplan-Meier method was used to analyse the **(A)** overall survival and **(B)** progression-free survival probability according to STn expression, with statistical significance determined using the log-rank test. The frequency distribution of cases **(C)** with age ≥ 62 years and < 62 years old, **(D)** with tumour size ≥ 25 mm and < 25 mm, **(E)** with grade III and grade I/II, and **(F)** with or without metastasis between the STn- and STn + groups was analysed with the Fisher exact test.

Survival analysis revealed that STn + patients exhibited a significantly reduced survival relative to the STn- group, showing 50% probability of survival 7 years post-diagnosis (Fig. 1A). Furthermore, a nearly significant decreased progression-free survival was observed for the STn + patients (Fig. 1B). Furthermore, the STn + was associated with increased age and was also marginally associated with increased tumour size (Fig. 1C and D), and tumours with a higher grade (grade III) were significantly more prevalent in the STn- group (Fig. 1E). Interestingly, within the STn + group, higher grade tumours were more prominent in comparison to lower grade (Fig. 1E). The percentage of cases that developed metastasis was higher in the case of the STn + group (~ 60 vs. ~ 40%), but no significant correlation was identified (Fig. 1F).

**Fig. 2:**
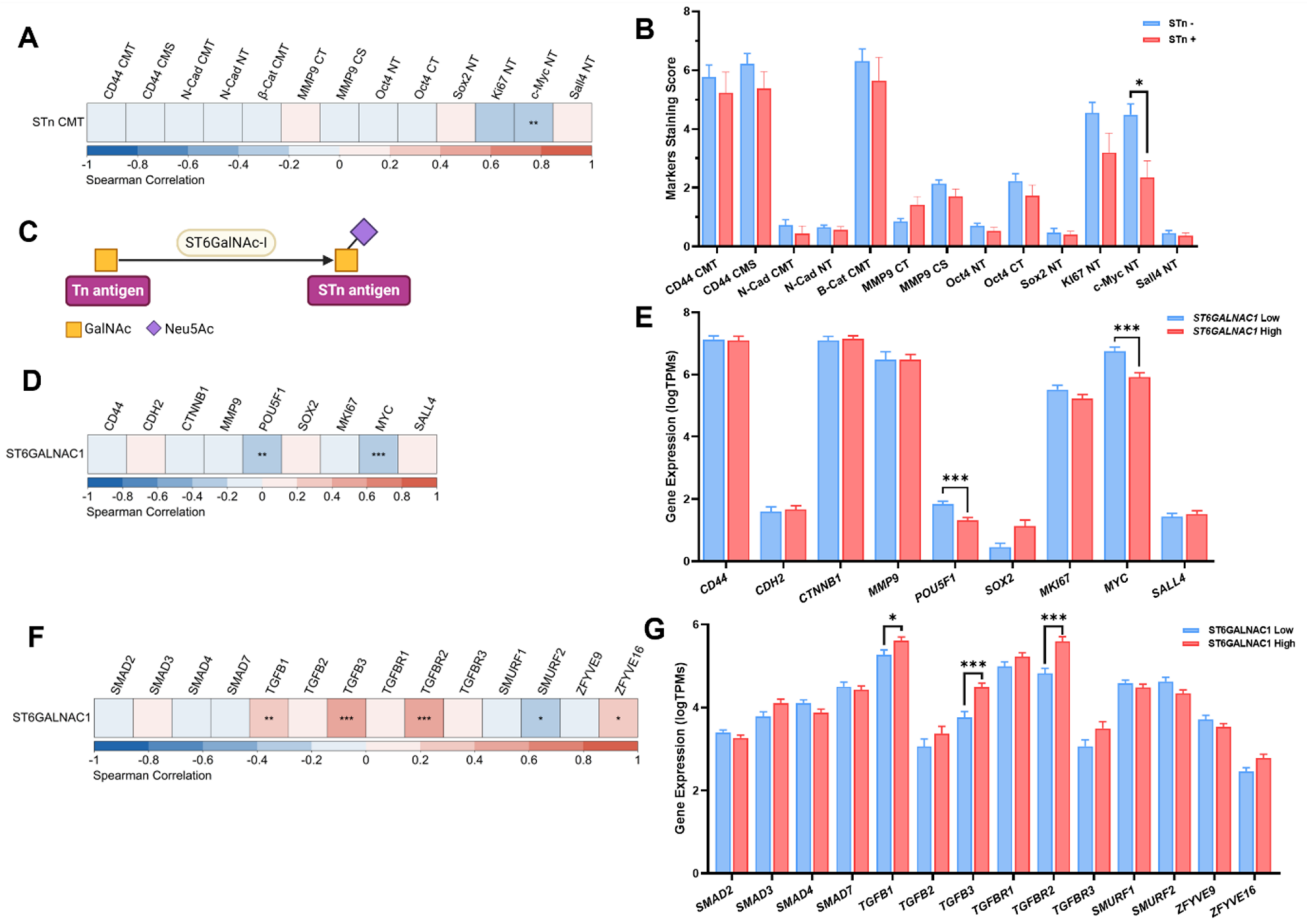
The STn antigen and *ST6GALNAC1* expression are inversely related to the c-Myc biomarker and *MYC* gene and positively associated with the TGF-β pathway genes in TNBC. The relationship between the STn and other biomarkers accessed was analysed **(A)** with the Spearman correlation analysis, with adjusted p-value (adj.p-value) by the False Discovery Rate (FDR) method, represented as heatmap distribution, and **(B)** with the comparison of the expression of the biomarkers between STn- and STn + groups, analysed using the Wilcox t-test with FDR adj.p-value. **C)** The STn is formed by the addition of a sialic acid (Neu5Ac) by the ST6GalNAc-I enzyme to the Thomson-nouveau (Tn) antigen. The Tn is composed of an N-acetylgalactosamine (GalNac) linked to a serine or threonine residue of a protein (non-represented). The relationship between the *ST6GALNAC1* gene expression and the other biomarkers genes was also accessed **(D)** with the Spearman correlation analysis, with FDR adj.p-value and **(E)** with the comparison of the biomarker’s gene expression between *ST6GALNAC1* Low and *ST6GALNAC1* High expression groups using the Wilcoxon t-test with FDR adj.p-value. The relationship between the *ST6GALNAC1* gene expression and the TGF-β pathway genes was evaluated **(F)** with the Spearman correlation analysis, with FDR adj.p-value and **(G)** with the comparison of the TGF-β pathway genes expression between *ST6GALNAC1* Low and *ST6GALNAC1* High expression groups using the Wilcoxon t-test with FDR adj.p-value. Data represented in the bar plots is expressed as the mean values ± SEM. N-Cad: N-Cadherin; β-Cat: β-Catenin; CMT: Cytoplasm and membrane in the tumour; CMS: Cytoplasm and membrane in the stroma; NT: Nucleus in the tumour; CT: Cytoplasm in the tumour; CS: Cytoplasm in the stroma; *CDH2*: N-Cadherin gene; *CTNNB1*: β-Catenin gene; *POU5F1*: Oct4 gene; * *adj.p* < 0.05, ** *adj.p* < 0.01, *** *adj.p* < 0.001.

To further explore the STn role in TNBC, we assessed its correlation with the cancer-related biomarkers also evaluated with IHC. Indeed, we detected a significant negative correlation between STn and the c-Myc protein (Fig. 2A and Supplementary Table S4). Concordantly, c-Myc was significantly reduced in the STn + group (Fig. 2B and Supplementary Table S5), indicating a possible link between STn presence and c-Myc expression.

We decided to corroborate our findings in an independent cohort enclosing 160 patients, the TCGA-TNBC. Here, we used as STn surrogate the expression levels of *ST6GALNAC1* gene, encoding the enzyme ST6GalNAc-I responsible for the synthesis of STn (Fig. 2C). No significant differences were observed in survival between the *ST6GALNAC1* high and low groups (Supplementary Fig. S3A/B). However, a significant negative correlation was detected between the expression of the *ST6GALNAC1* and two cancer-related biomarkers genes: *MYC* and *POU5F1* (Fig. 2D and Supplementary Table S6). Concordantly, significantly lower levels of the *MYC* and *POU5F1* genes were observed in the high *ST6GALNAC1* group (Fig. 2E and Supplementary Table S7).

Since the transforming growth factor beta (TGF-β) signalling pathway regulates *MYC* gene expression, we investigated for correlations between the *ST6GALNAC1*, and genes involved in this pathway. A positive correlation was found with the *TGFB1*, *TGFB3*, TGF-β Receptor 2 (TGFBR2) and the *ZFYVE16* gene and a negative correlation with the *SMURF2* gene (Fig. 2F and Supplementary Table S8). Furthermore, the *TGFB1*, *TGFB3*, and *TGFBR2* genes showed higher expression in the high *ST6GALNAC1* group (Fig. 2G and Supplementary Table S9).

### ST6GALNAC1 expression in TNBC is associated with specific immune signalling pathways and cell infiltration

**Fig. 3:**
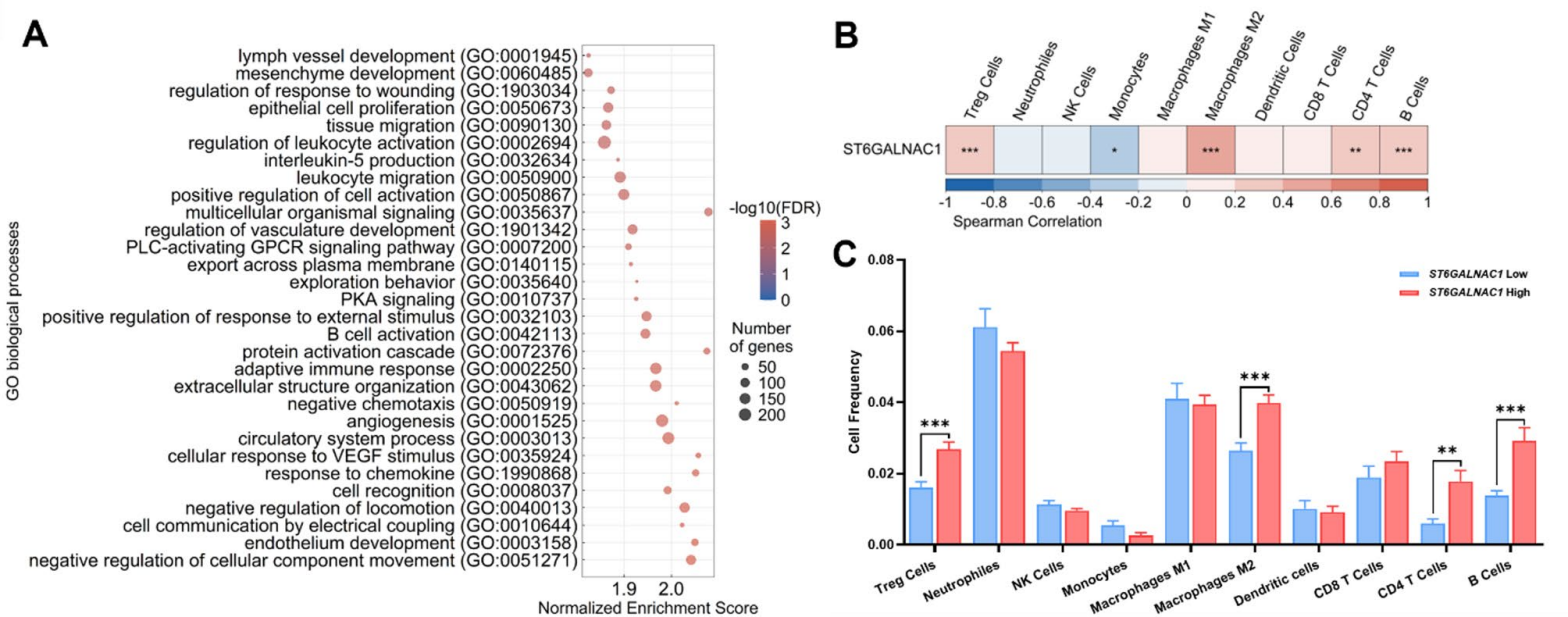
Immune-related features associated with *ST6GALNAC1* expression levels in the TCGA-TNBC cohort. **(A)** Representative plot with the normalised enrichment score of the top 30 positive statistically significant (FDR < 0.05) non-redundant enriched terms related to Gene Ontology (GO) biological processes. The GO terms were obtained with the gene set enrichment analysis (GSEA) performed using the WebGestalt platform. The relationship between the *ST6GALNAC1* gene expression and the immune cells frequency was analysed with the **(B)** Spearman correlation analysis, with adjusted p-value (adj.p-value) by the False Discovery Rate (FDR) method, represented as heatmap distribution, and with the **(C)** comparison of the immune cells frequency between *ST6GALNAC1* Low and *ST6GALNAC1* High expression groups using the Wilcox t-test with FDR adj.p-value. Data is expressed as the mean values ± SEM. Immune cell frequency was obtained through The Cancer Immune Atlas (TCIA) database. Treg: regulatory T cells; NK: Natural Killer Cells. * adj.*p* < 0.05, ** adj.*p* < 0.01, *** adj.*p* < 0.001.

Comparing the transcriptome profiles between TCGA-TNBC patients with high and low *ST6GALNAC1* expression, we obtained 1300 differentially expressed genes (DEGs) with adj.p-value < 0.05 and logFC cut-off 1 (Supplementary Fig. S4 and Table S10). Gene set enrichment analysis (GSEA) identified revealed several biological processes in the 30 most significantly enriched linked to immune regulation, including leukocyte activation, migration, and IL-5 production (Fig. 3A, Supplementary Table S11).

Further, analysing the association between the *ST6GALNAC1* gene expression levels and the frequency of immune cell infiltrates (derived from RNAseq-based deconvolution), a positive significant correlation was found with the presence of macrophages M2, regulatory T (Tregs), CD4 T and B cells, and a negative with the monocytes (Fig. 3B Supplementary Table S12). Further, the macrophages M2, Tregs, CD4 T and B cells were higher in the tumours with high *ST6GALNAC1* expression (Fig. 3C Supplementary Table S13).

### STn expression in a TNBC in vitro model negatively associates with c-Myc protein expression and impacts cellular proliferation and macrophage polarization

**Fig. 4:**
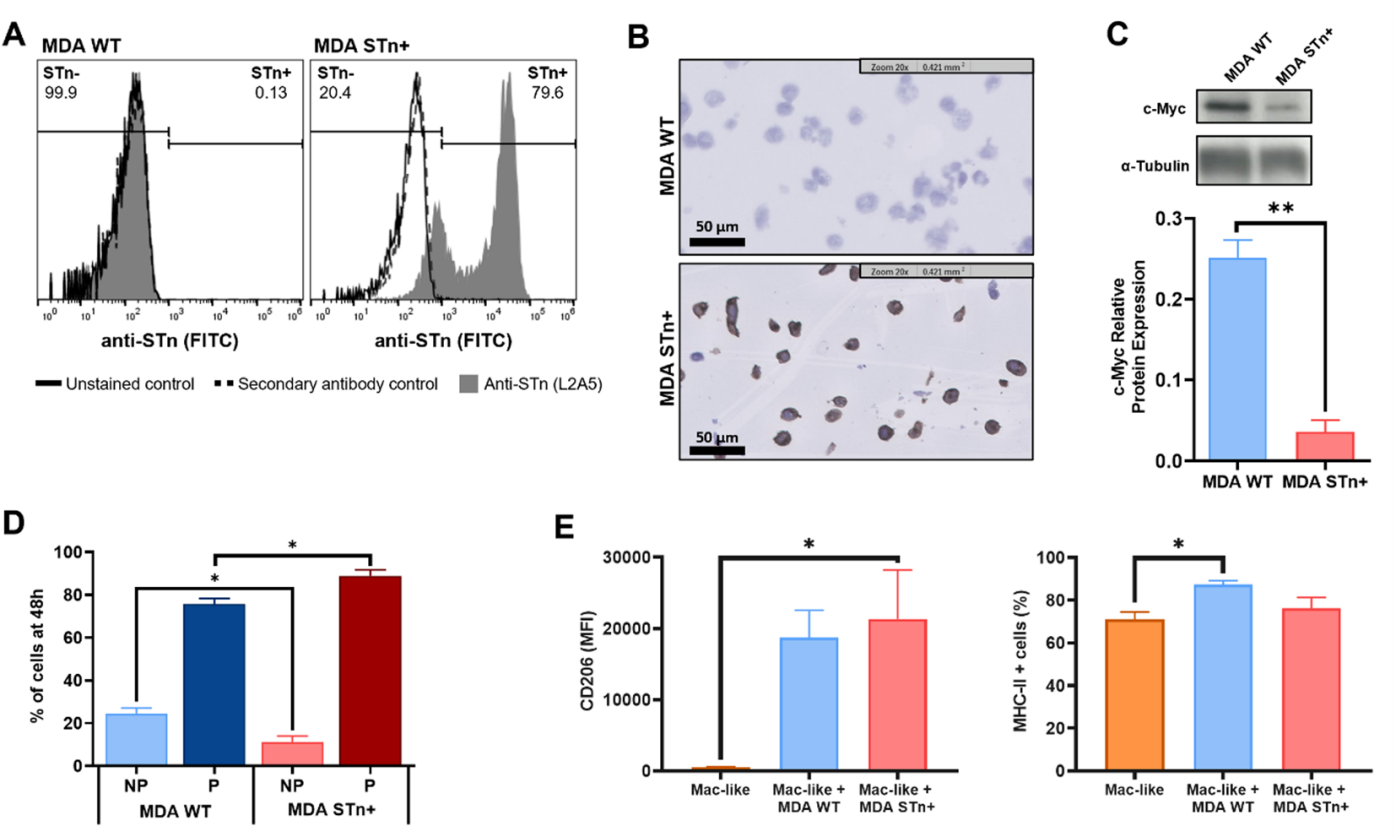
Characterization of TNBC cell line overexpressing the STn antigen. The expression of the STn in the MDA-MB-231 TNBC cell lines, transfected with an expression vector encoding the *ST6GALNAC1* gene (MDA STn+) and WT (MDA WT), was evaluated by **(A)** flow cytometry and **(B)** immunocytochemistry. **(C)** The c-Myc relative protein expression was evaluated by western blot, with α-Tubulin used as a loading control, and statistical significance was evaluated with the parametric two-tailed unpaired t-test with Welch’s correction (complete blot in Supplementary Fig. S7). **(D)** The proliferation capacity in both MDA cell lines was analysed by CFSE dilution assay after 48 h, and the non-parametric Mann-Whitney test was used. P is the proliferative cells, and NP is the non-proliferative. **(E)** PBMC-isolated monocytes were co-cultured with the MDA STn + and MDA WT cell lines for 5 days and analysed by flow cytometry for the mean fluorescence intensity (MFI) of the CD206 biomarker (left) and frequency of cells positive for the MHC-II biomarker (right). The parametric one-way ANOVA and Tukey’s multiple comparisons test were used. Results represent at least three independent experiments, and data is expressed as the mean values ± SEM. * *p* < 0.05, ** *p* < 0.01.

To further validate our findings, we used the TNBC cell line MDA-MB-231, genetically modified to stably overexpress *ST6GALNAC1* (overexpression confirmed by RT-qPCR in Supplementary Fig. S5) and consequent presence of STn antigen (MDA STn+; Fig. 4A and B, gating strategy in Supplementary Fig. S6), confirming the critical role of *ST6GALNAC1* in the STn expression. In accordance with HSJ-TNBC and TCGA-TNBC cohorts MDA STn + cells showed lower c-Myc protein expression compared to the WT (Fig. 4C, Supplementary Fig. S7). Furthermore, the CFSE dilution assay showed the MDA STn + cell line exhibited higher proliferation at 48 h compared to MDA WT cells, indicating a potential influence of STn expression in promoting cell proliferation (Fig. 4D).

To explore the link between STn and immune cells, primary monocytes were co-cultured with the MDA STn + and WT cells for 5 days to allow differentiation into macrophage-like cells. To assess their polarization status, we analysed markers representative of distinct macrophage phenotypes: MHC-II as a hallmark of pro-inflammatory, anti-tumoral M1 macrophages, and CD260 and CD163 as markers of anti-inflammatory, pro-tumoral M2 macrophages^39–41^. Macrophage-like cells cultured with MDA STn + cells showed a significant increase in the cell surface expression of the CD206 biomarker (Fig. 4E and Supplementary Fig. S8A), indicative of polarization towards an M2 macrophage phenotype, with no increase in the percentage of positive cells (Supplementary Fig. S8B). The percentage of cells positive for the MHC-II biomarker was significantly increased in the macrophage-like cells cultured with MDA WT but not with the STn + cells (Fig. 4E and Supplementary Fig. S8A), in agreement with polarization towards M1 macrophage phenotype, with no significant increase in its cell surface expression (Supplementary Fig. S8B). The CD163 was increased in the macrophage-like cells in co-culture compared to the macrophages alone, with no difference between the co-culture with the MDA STn + or WT (Supplementary Fig. S8B).

## Discussion

TNBC is an aggressive type of breast cancer with elevated metastatic potential, recurrence, and poor prognosis^42^. This study provides a novel and comprehensive characterization of TNBC tumours and reports for the first time a ~ 25% TNBC subtype expressing STn with a poorer prognosis. For a robust investigation, we used two patient cohorts, and a complementary approach analysing immunohistochemistry, gene expression, clinical approaches, and in vitro cell culture experiments data.

Our study underscores the significant diversity among TNBC tumours concerning biomarker expression. The observed survival differences in the HSJ-TNBC and TCGA-TNBC cohorts highlights the complex nature and molecular landscape of TNBC, emphasising the need for personalised approaches to redefine diagnosis and treatment. Of note, we assumed a strong link between STn expression analysed in the HSJ-TNBC cohort and *ST6GALNAC1* expression analysed in the TCGA-TNBC cohort, as ST6GalNAc-I, is the primary sialyltransferase driving STn biosynthesis^9^. While additional factors such as GalNAc transferases (GALNTs) activity and the loss of core 1 β1,3-galactosyltransferase (C1GalT1) or its chaperone Cosmc can also influence STn formation, evidence from overexpression models (including the one used on this study) supports ST6GalNAc-I as the enzyme most directly associated with STn^17,29,43^.

We identified a subpopulation of TNBC expressing STn, which is associated with increased age and tumour size, and exhibited significantly poorer survival outcomes, consistent with prior studies in breast and other cancers^19,44,45^. Although there was a trend for STn to be more prevalent in cases with higher grade and those that developed metastasis, no statistically significant association with metastasis was found in our cohorts. The limited sample size may have constrained the statistical power of these analyses, and it is plausible that a larger cohort could reveal a more robust association. This evidences STn as a possible prognostic factor for worst disease outcomes and highlights its role in TNBC, which has remained unexplored until now. Importantly, all STn⁺ cases in our cohort exhibited membrane staining, which is therapeutically relevant, although most also showed additional cytoplasmic signal. This reflects the biosynthetic pathway of STn and agrees with previous observations in breast cancer cells and tissues^19,46,47^. Interestingly, the percentage of STn + cases was similar to the subsets of TNBC expressing the transcription factors Sox2 and Sall4, which have been considered undruggable due to their nature as transcription factors, posing a significant challenge. Therefore, STn as a cell surface biomarker might bring a new opportunity for treatment.

In the understanding of STn in tumorigenesis, we found a negative association between STn and the c-Myc biomarker despite a tendency to be associated with increased tumours and poor prognosis. This negative correlation was further observed at the transcriptomic level between the *ST6GALNAC1* gene, responsible for STn synthesis, and the *MYC* gene^9,10,29^. In addition, the MDA-MB-231 cell line overexpressing STn, derived from overexpression of the *ST6GALNAC1* gene, also revealed lower levels of c-Myc protein and higher proliferation rates, confirming STn’s association with deregulated c-Myc. While c-Myc is a known powerful oncogene linked to stem cell features, overexpressed in various cancers, its role is complex^48,49^. The *MYC* gene expression is regulated by the TGF-β signalling pathway, which initiates with the TGF-β ligands binding to the TGFR-2 and forming a complex with TGFR-1, activating the kinase that phosphorylates Smad2 and 3 proteins that complex with Smad4 and translocate into the nucleus and regulate gene expression, reducing the *MYC* gene expression^50–52^. While c-Myc inhibition may suppress cell division, it can also promote tumour cell survival and resistance to apoptosis, allowing cancer cells to evade cell death signals induced by therapies^53^. We found a positive correlation with the genes *TGFB1*, *TGFB3*,* TGFBR2 and ZFYVE16*, coding for TGF-β ligands, receptor, and the endofin protein involved in the Smad2/3-Smad4 complex formation, and a negative correlation with the *SMURF2* gene responsible for ubiquitination and proteasomal degradation of the TGF-β receptors, an essential negative regulator of TGF-β signalling^54,55^. These results show a positive link between the increase of *ST6GALNAC1*, consequent STn expression, and the activation of the TGF-β pathway, explaining the reduced expression of the c-Myc biomarker in our models.

TGF-β is a key regulator of cell behaviour, and mutations or alterations in its pathway can promote immune evasion, leading to the recruitment of suppressive myeloid cells, such as macrophages, which promote metastasis^56,57^. Reduced c-Myc expression may deregulate gene transcription, allowing cells to escape immune surveillance. Our findings highlight and add STn to the complex interplay between TGF-β signalling, c-Myc expression, and tumour development.

The TCGA-TNBC cohort tumours revealed a correlation between the *ST6GALNAC1* and macrophages M2 and Tregs, which exert pro-tumorigenic effects through the release of cytokines and chemokines, such as IL-10 and TGF-β, that support tumour growth and promote an immune suppressive environment and consequent immune evasion^58,59^. The secretion of IL-10 by the macrophages M2 and Treg cells drives the differentiation of Th2 cells, potentially elucidating the observed increase in CD4 + T cells. This subset releases cytokines such as IL-4 and IL-13, further promoting the M2 phenotype^60^. Even though an increase in B cells is observed, these may not effectively mount anti-tumour immune responses due to the overall immunosuppression mediated by M2 macrophages and Tregs.

In this study, we showed that macrophages co-cultured with the MDA STn + cell lines showed a significant CD206 increase, while co-cultured with the WT cell line favoured higher proportion of MHC-II positive cells. This aligns with previous reports that STn is recognized by CD206 which can drive an immunosuppressive macrophage phenotype through the induction of IL-10 and downregulation of pro-inflammatory mediators^61,62^. The observation that CD163 was upregulated in both co-culture conditions suggests that tumour-macrophage interactions broadly favour M2 polarization, although STn expression appears to selectively enhance the CD206 axis. These findings are consistent with observations in other tumour types. For instance, in bladder cancer, elevated expression of *ST6GALNAC1* correlated with increased immature dendritic cells (DCs) unresponsive to further maturation stimuli and low levels of Th1-inducing cytokines IL-12 and TNF-α, resulting in T cells exhibiting a phenotype indicative of tolerance. Furthermore, locking the STn antigens reduced tolerance induction and promoted DC maturation^17^. Importantly, in a recent study, a recombinant glycan-binding receptor CD301 (also known as macrophage galactose-type lectin, MGL) – a C-type lectin receptor, commonly expressed on macrophages – was found to recognize the Tn and STn antigens in breast cancer samples^63^. Specifically, in TNBC samples, high staining frequencies of these glycan structures recognized by CD301 were significantly associated with worse prognosis, reinforcing our results^63^. Furthermore, engagement of CD301 with its ligands has been shown to increase IL-10 secretion and promote apoptosis of effector T cells, thereby supporting an immunosuppressive microenvironment^64,65^. These observations resonate with our findings, where STn⁺ tumour cells selectively promoted CD206 upregulation on macrophages, highlighting a converging role of STn antigens in fostering an immune-suppressive phenotype through multiple glycan–C–type lectin receptor axes, including both CD206 and CD301.

Together, these results underscore the significant role of the STn antigen in promoting an immunosuppressive environment within TNBC tumours, which contributes to immune evasion and poorer disease outcomes. This highlights the potential of STn as a crucial biomarker in TNBC, paving the way for targeted therapeutic interventions and more personalised treatment strategies in managing this aggressive cancer subtype.

## Supplementary Information

Below is the link to the electronic supplementary material.

Supplementary Material 1

Supplementary Material 2

## Data Availability

The dataset generated during and/or analysed during the current study that supports the findings related to the HSJ-TNBC cohort are available in Supplementary Table S2 of this article. The data that support the findings related to the TCGA-TNBC cohort here are in whole or part based upon data generated by the TCGA Research Network and openly available at [https://www.cancer.gov/tcga](https:/www.cancer.gov/tcga) . The *in vitro* data that support the findings of this study are available from the corresponding author, p.videira@fct.unl.pt, upon reasonable request.

## Code Availability

All the code developed and needed to reproduce the analyses is available at: https://gitfront.io/r/RitaAL/KKzGU7s4R54V/STn-in-TNBC/.
