# Supplementary material for: Sialyl-Tn expression correlates with reduced c-Myc and immune modulation in triple negative breast cancer
Source: Sci Rep. 2025 Oct 28;15:37615. doi: 10.1038/s41598-025-21496-3 (PMC12569379; doi:10.1038/s41598-025-21496-3)
Supplement: Supplementary file 2 — Supplementary Material 2 [file 41598_2025_21496_MOESM2_ESM.docx]

**Sialyl-Tn expression correlates with reduced c-Myc and immune modulation in Triple Negative Breast Cancer**

Rita Adubeiro Lourenço^1,2^, Daniela Ferreira Barreira^1,2,3^, Carla Lopes^4^, Pedro Granjo­­­^1,2,3^, Ana Sofia Rodrigues^1,2,3^, Zélia Silva^1,2,3^, Manuela Martins^4^, Ana Rita Grosso^1,2^, Paula A Videira^1,2,3*^

^1^ UCIBIO – Applied Molecular Biosciences Unit, Department of Life Sciences, NOVA School of Science and Technology | FCT NOVA, Universidade NOVA de Lisboa, 2829-516 Caparica, Portugal

^2^ Associate Laboratory i4HB - Institute for Health and Bioeconomy, NOVA School of Science and Technology | FCT NOVA, Universidade NOVA de Lisboa, 2829-516 Caparica, Portugal

^3^ CDG & Allies – Professionals and Patient Associations International Network (CDG & Allies – PPAIN), 2829-516 Caparica, Portugal

^4^ Centro Hospitalar Universitário de Lisboa Central, EPE e Serviço de Anatomia Patológica 1150-199, Lisboa, Portugal

*Corresponding author: [p.videira@fct.unl.pt](mailto:p.videira@fct.unl.pt)

**Authors e-mail address:**

Rita A Lourenço: [ritaalourenco95@gmail.com](mailto:ritaalourenco95@gmail.com)

Daniela F Barreira: [ds.barreira1@gmail.com](mailto:ds.barreira1@gmail.com)

Carla Lopes: [carlabritolopes@gmail.com](mailto:carlabritolopes@gmail.com)

Pedro Granjo: [pmgranjo@gmail.com](mailto:pmgranjo@gmail.com)

Ana S Rodrigues: [anasofiarodrigues3@gmail.com](mailto:anasofiarodrigues3@gmail.com)

Zélia Silva: [zm.silva@fct.unl.pt](mailto:zm.silva@fct.unl.pt)

Manuela Martins: [mmanuelacmartins@gmail.com](mailto:mmanuelacmartins@gmail.com)

Ana R Grosso: [ar.grosso@fct.unl.pt](mailto:ar.grosso@fct.unl.pt)

**Supplementary Figures**


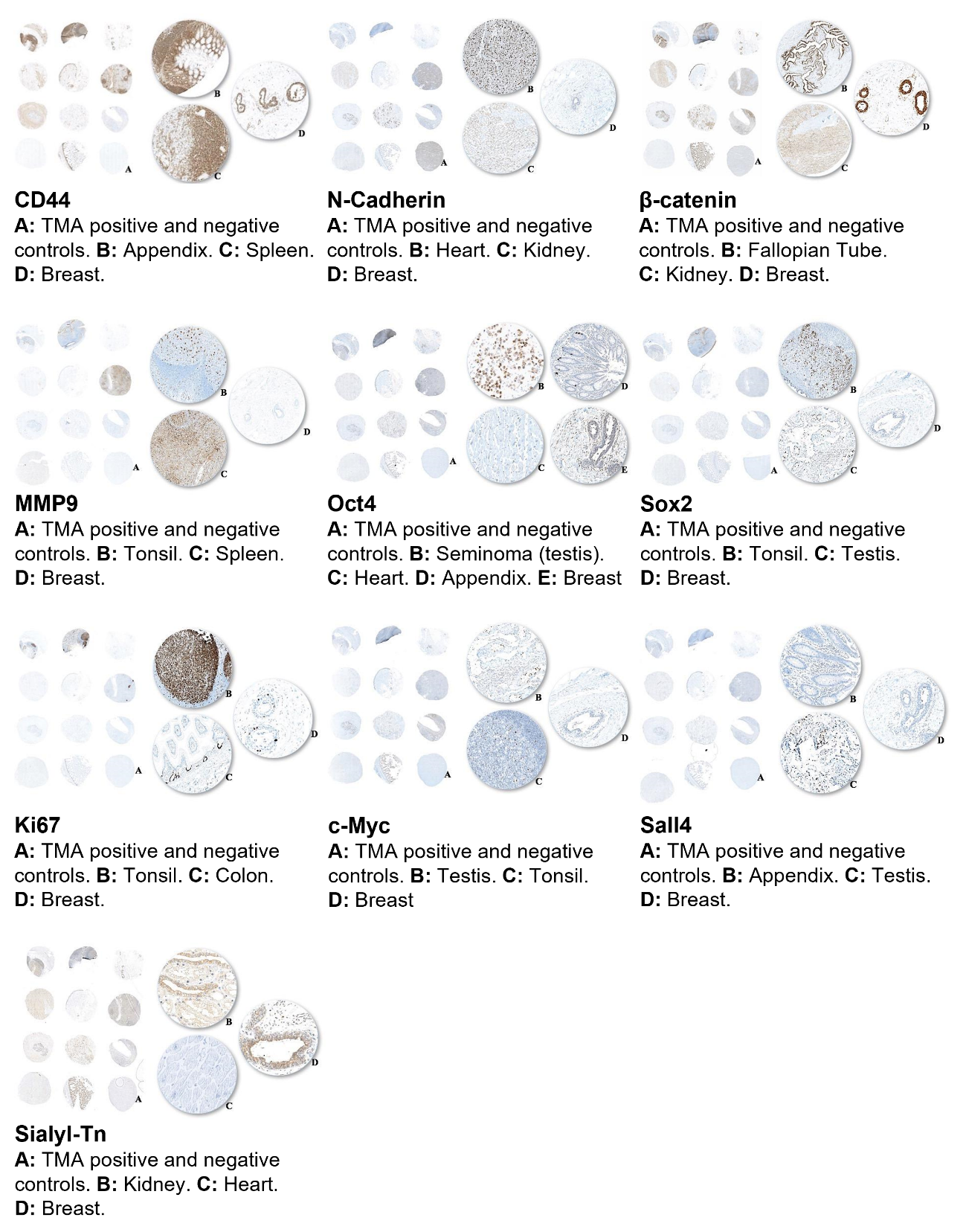
**Supplementary Figure S1. Immunohistochemistry staining optimization.** All antibodies were tested with the appropriate positive and negative controls, selected according to the Human Protein Atlas indication. Maximum immunostaining intensity was defined as the maximum intensity observed in the stained positive controls. TMA: Tissue Microarray.

**
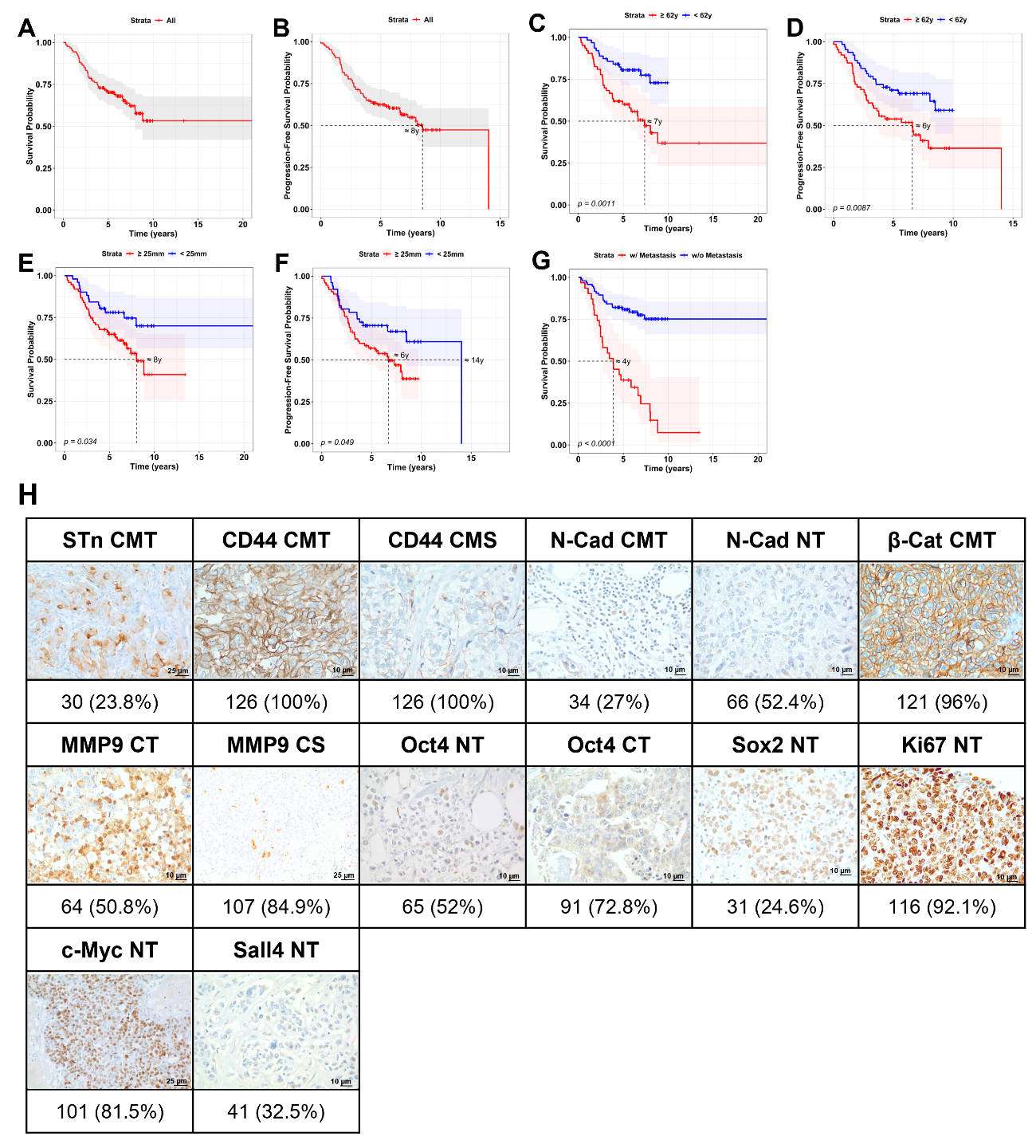
**

**Supplementary Figure S2.** **Characterization of the HSJ-TNBC cohort population.** Tumour samples were collected from 126 women with TNBC and the clinical data available for the HSJ-TNBC Cohort was used to assess the **(A)** overall survival and **(B)** progression-free survival probability since diagnosis using the Kaplan-Meier method. The overall and progression-free survival probability according to **(C, D)** age**,** **(E, F)** tumour size and **(G)** overall survival according to metastasis were also analysed using the Kaplan-Meier method with statistical significance determined with the log-rank test. Tumour samples were studied for biomarker expression with immunohistochemistry of tissue microarrays (TMA) and in **(H)** are representative cases demonstrating the staining of each biomarker (40x magnification, except STn and c-Myc 20x magnification). Under each biomarker is the number of samples with a staining score ≥ 1 and the percentage it represents in the cohort (N=126). Oct 4 NT and CT and c-Myc NT had one and two fewer samples interpreted, respectively, due to tissue loss in the microtomy. N-Cad: N-Cadherin; β-Cat: β-Catenin; CMT: Cytoplasm and membrane in the tumour; CMS: Cytoplasm and membrane in the stroma; NT: Nucleus in the tumour; CT: Cytoplasm in the tumour; CS: Cytoplasm in the stroma.


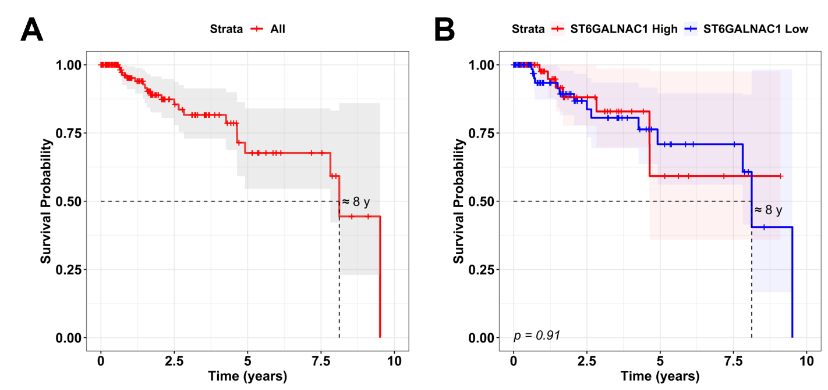
**Supplementary Figure S3. Overall survival analysis of the TCGA-TNBC Cohort.** The Kaplan-Meier method was used to assess the overall survival **(A)** and the overall survival probability according to *ST6GALNAC1* expression **(B)**, with statistical significance determined using the log-rank test.


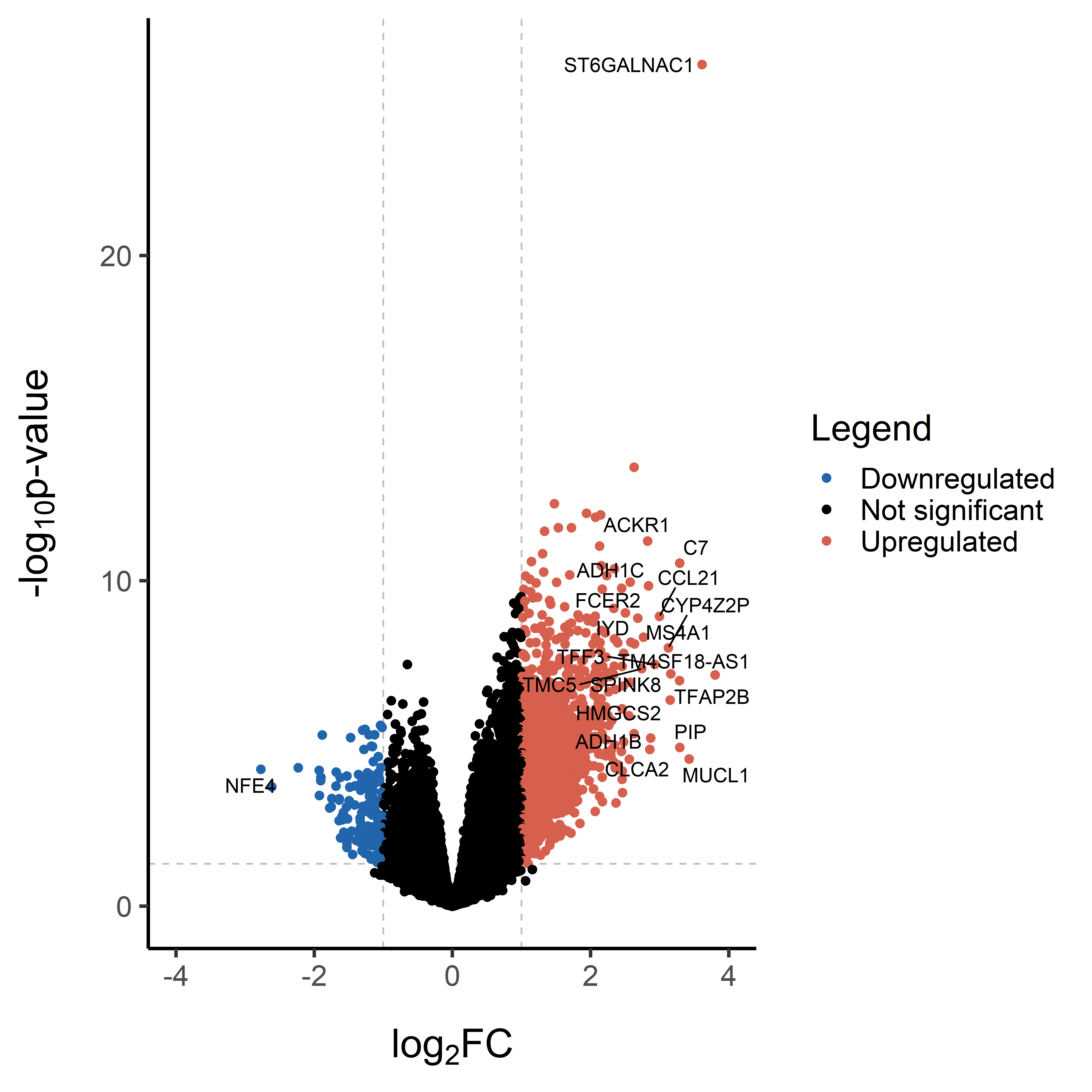


**Supplementary Figure S4. Differently expressed genes (DEGs) between *ST6GALNAC1* high and low expression groups.** Volcano plot representing the log fold change (log_2_FC) and statistical significance (-log_10_p-value) of the DEGs between *ST6GALNAC1* high and low expression groups. Represented are the 20 most significant genes (p < 0.05) and with a log_2_FC cut-off of 1.

**
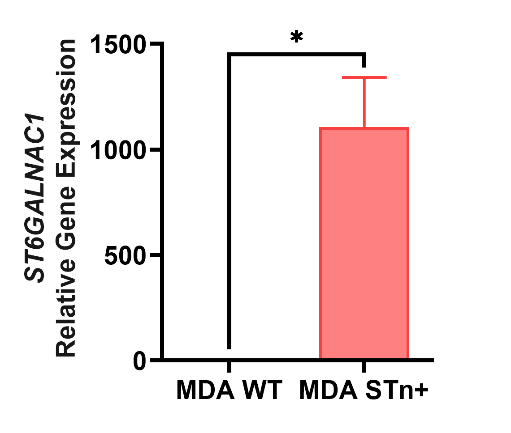
Supplementary Figure S5.** **Expression of the *ST6GALNAC1* gene in the MDA-MB-231 WT and STn+ cell lines.** The MDA-MB-231 TNBC cell line was stably transfected with an expression vector encoding the ST6GalNAc1 enzyme to obtain a TNBC model overexpressing the STn antigen. To quantify the expression of the *ST6GALNAC1* gene, total RNA was extracted followed by reverse transcription-quantitative polymerase chain reaction (RT-qPCR), as described previously (DOI: 10.1002/1878-0261.12163). The parametric two-tailed unpaired t-test with Welch’s correction was used to compare. * p value < 0.05.


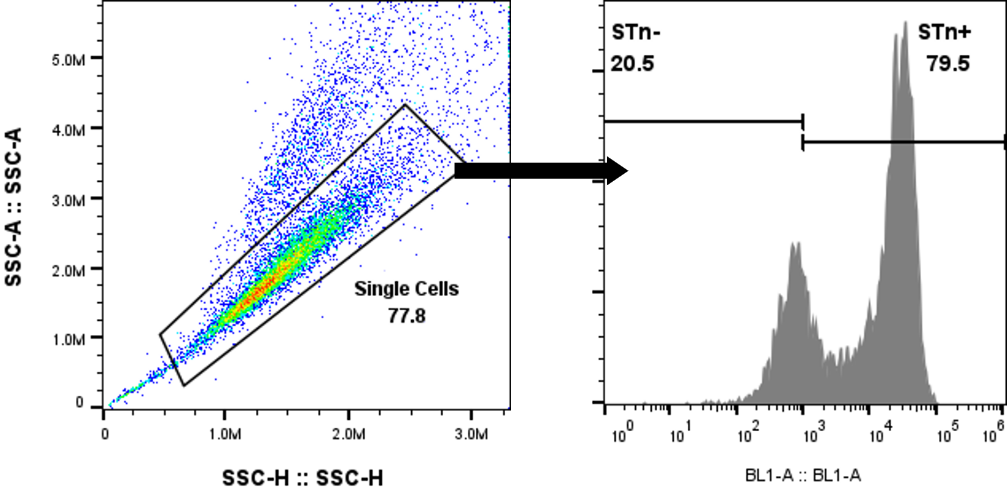


**Supplementary Figure S6. Gating strategy for the analysis of the STn expression in the MDA-MB-231 STn+ and WT cell lines.** The presence of STn was assessed in both the MDA STn+ and MDA WT cell lines through flow cytometry. The plots are representative of the MDA STn+ cell line.


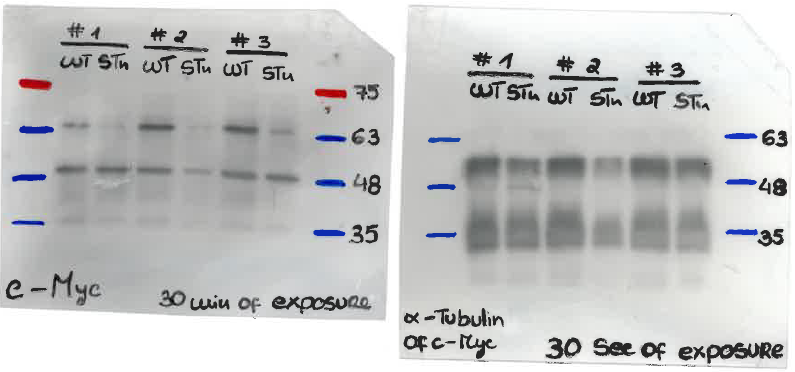

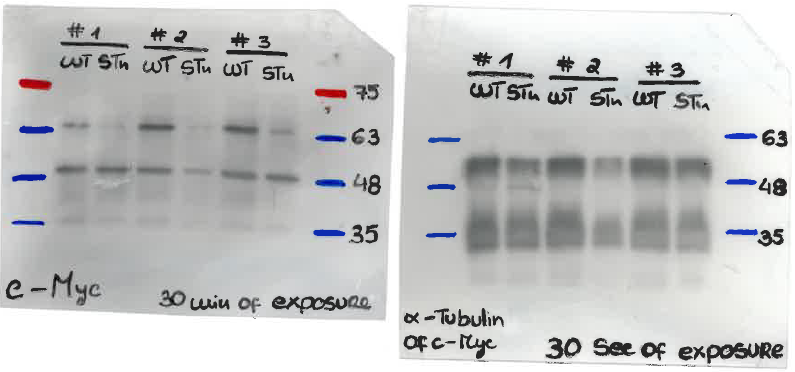


**A**

**B**

**Supplementary Figure S7. Whole Western blot films.** Here is shown all bands and molecular weight markers of c-Myc (~65 kDa) staining (A) and α-tubulin (~50 kDa) staining (B). Lanes #1, #2 and #3 correspond to n=1, n=2 and n=3, respectively. WT: MDA-MB-231 WT; STn: MDA-MB-231 STn+.


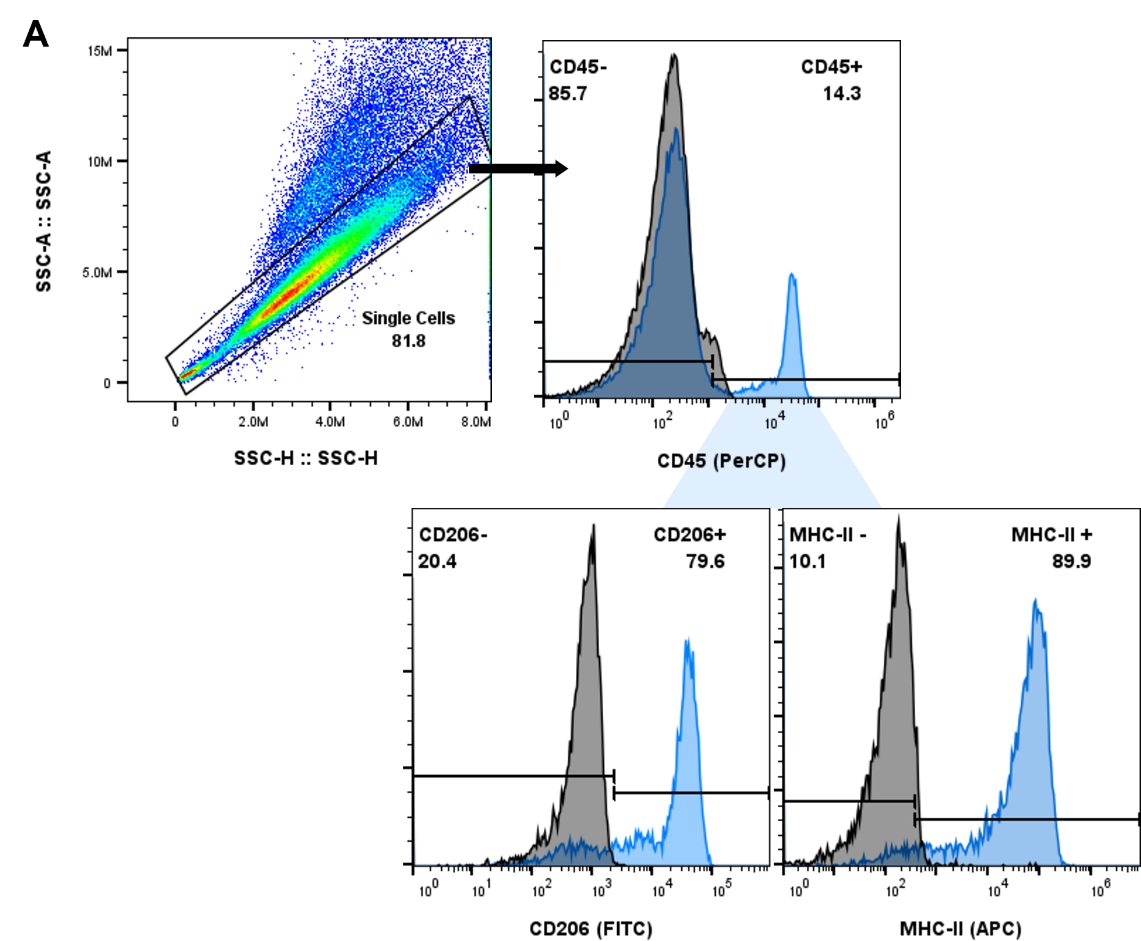

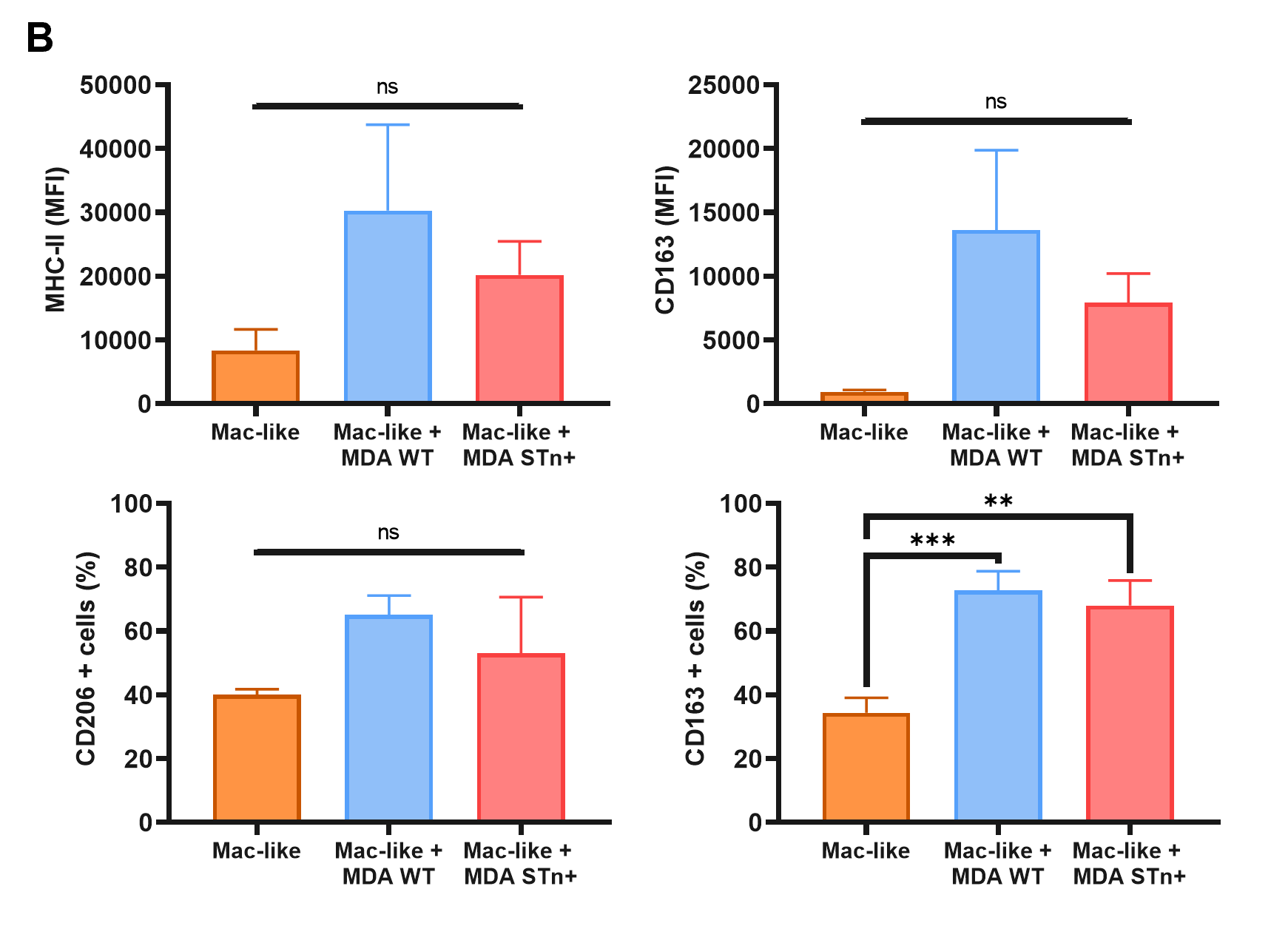


**Supplementary Figure S8. Analysis of the CD206, CD163 and MHC-II in the macrophage-like monocytes.** The monocytes isolated from PBMCs were co-cultured with the MDA-MB-231 WT and STn+ cell lines, or cultured alone, for 5 days to allow for adherence and differentiation. The macrophage-like cells were evaluated for the expression of the CD206, CD163 and MHC-II molecules trough flow cytometry. To select the immune cells from the co-culture, the CD45 immune biomarker was used. The gating strategy used to select the CD45+ and analysis of the other biomarkers is depicted with representative plots from one sample in **(A)**. The mean fluorescence intensity (MFI) of the MHC-II and CD163, and frequency of cells positive for the CD206 and CD163 is depicted in the two top and two bottom plots, respectively, in **(B)**. The parametric one-way ANOVA and Tukey’s multiple comparations test was used. Results represent at least three independent experiments, and data is expressed as the mean values ± SEM. ***p* < 0.01, ******* *p* < 0.001.
